# Trial registration and selective outcome reporting in 585 clinical trials investigating drugs for prevention of postoperative nausea and vomiting

**DOI:** 10.1186/s12871-021-01464-w

**Published:** 2021-10-19

**Authors:** Manuel Riemer, Peter Kranke, Antonia Helf, Debora Mayer, Maria Popp, Tobias Schlesinger, Patrick Meybohm, Stephanie Weibel

**Affiliations:** 1 Department of Anaesthesiology, Intensive Care, Emergency and Pain Medicine, University Hospital Wuerzburg, Oberduerrbacher Str. 6, 97080 Wuerzburg, Germany; 2 Department of Surgery, Kantonsspital Glarus, Glarus, Switzerland

**Keywords:** Clinical trial, Postoperative nausea and vomiting, Selective outcome reporting, Systematic review, Trial registration

## Abstract

**Background:**

Selective outcome reporting in clinical trials introduces bias in the body of evidence distorting clinical decision making. Trial registration aims to prevent this bias and is suggested by the International Committee of Medical Journal Editors (ICMJE) since 2004.

**Methods:**

The 585 randomized controlled trials (RCTs) published between 1965 and 2017 that were included in a recently published Cochrane review on antiemetic drugs for prevention of postoperative nausea and vomiting were selected. In a retrospective study, we assessed trial registration and selective outcome reporting by comparing study publications with their registered protocols according to the ‘Cochrane Risk of bias’ assessment tool 1.0.

**Results:**

In the Cochrane review, the first study which referred to a registered trial protocol was published in 2004. Of all 585 trials included in the Cochrane review, 334 RCTs were published in 2004 or later, of which only 22% (75/334) were registered. Among the registered trials, 36% (27/75) were pro- and 64% (48/75) were retrospectively registered. 41% (11/27) of the prospectively registered trials were free of selective outcome reporting bias, 22% (6/27) were incompletely registered and assessed as unclear risk, and 37% (10/27) were assessed as high risk. Major outcome discrepancies between registered and published high risk trials were a change from the registered primary to a published secondary outcome (32%), a new primary outcome (26%), and different outcome assessment times (26%). Among trials with high risk of selective outcome reporting 80% favoured at least one statistically significant result. Registered trials were assessed more often as ‘overall low risk of bias’ compared to non-registered trials (64% vs 28%).

**Conclusions:**

In 2017, 13 years after the ICMJE declared prospective protocol registration a necessity for reliable clinical studies, the frequency and quality of trial registration in the field of PONV is very poor. Selective outcome reporting reduces trustworthiness in findings of clinical trials. Investigators and clinicians should be aware that only following a properly registered protocol and transparently reporting of predefined outcomes, regardless of the direction and significance of the result, will ultimately strengthen the body of evidence in the field of PONV research in the future.

**Supplementary Information:**

The online version contains supplementary material available at 10.1186/s12871-021-01464-w.

## Background

It is imperative to report results of clinical research on patients transparently, completely and not selectively. Non-publication of trials and selective reporting of outcome results, termed as publication bias [1] and selective reporting bias [2], respectively, can distort the evidence available for clinical decision making.

A cornerstone in ensuring transparency of clinical research and accountability in the planning, conduct and reporting of clinical trials is the introduction of trial registers [3]. Prospective registration of trials, which means registration before enrolment of patients, can protect not only against non-publication of trials, but also against selective reporting of outcome results. Selective decisions on outcome reporting are frequently driven by the statistical significance of outcome results and can lead to a change, introduction, or omission of at least one primary outcome [4]. Selective reporting of outcomes in published clinical trials occurred in 40 to 62% of studies [5].

Another crucial step towards improving transparency is provided by the International Committee of Medical Journal Editors (ICMJE), which has announced in 2004 that journals should require, as a precondition of publication, prospective registration in a public trials registry [6]. The full advantages of registration can only be achieved when trials are fully registered including all 20 items recommended in the WHO Minimum Trial Registration Data Set [7]. Finally, the AllTrials initiative (All Trials Registered | All Results Reported) was launched in January 2013 to draw attention to the issue of unreported trial data. It calls for all past and present clinical trials to be registered and their results reported (http://www.alltrials.net).

Despite all these movements towards complete transparency in registering and reporting of clinical studies, Al-Durra et al. showed that among all RCTs published in PubMed indexed journals in 2018 and registered in any WHO trial registry only 42% complied with prospective trial registration [8]. Trials in anaesthesiology research are registered less often and mostly inadequate, i.e. after the first patient was enrolled into the study and without a clearly defined primary outcome [9]. To the best of our knowledge, there is no large study investigating trial registration and selective outcome reporting in trials published in a specific field of clinical anaesthesia research in a broad range of different journals.

In this study, we analysed the 585 RCTs included in the recently published Cochrane review on drugs for prevention of postoperative nausea and vomiting (PONV) [10, 11] in terms of trial registration and selective outcome reporting. The underlying Cochrane Review was performed by the same study group that conducted this study. We aim to identify current limitations in trial registration and reporting of outcomes in PONV trials and to draw attention of clinical trial authors to these important issues when planning and conducting their future studies.

## Methods

This retrospective study was part of a recently published Cochrane systematic review with network meta-analysis on antiemetic drugs that was registered in the Cochrane Database of Systematic Reviews [10, 11], (PROSPERO CRD42017083360). This study examines the identical study set as included in the Cochrane systematic review.

### Eligibility criteria for article selection and information sources

The Cochrane review included RCTs that were reported as full-text publication in any journal or as comprehensive clinical study report, published in any language. Studies investigated adult participants undergoing any type of surgery with general anaesthesia; and compared single or multiple pharmacological intervention(s) with antiemetic action belonging to one of the six drug classes (5-HT_3_ receptor antagonists, D_2_ receptor antagonists, NK_1_ receptor antagonists, corticosteroids, antihistamines (histamine 1 receptor antagonists), and anticholinergics) versus each other, versus no treatment, or versus placebo.

We searched the Cochrane Central Register of Controlled Trials (CENTRAL), MEDLINE, Embase, CINAHL, study registers (ClinicalTrials.gov, WHO ICTRP), and the reference lists of relevant systematic reviews for eligible trials in November 2017. Details of the search strategy are provided in the Cochrane review [10, 11].

### Study selection and data extraction

The review team independently, and in duplicate, assessed trials for inclusion and extracted data. In brief and relevant for this study on selective outcome reporting, we extracted the trial registration number, the start (date of first participant’s enrolment) and duration of the study, and study’s outcomes with details. We have defined a primary outcome as that which was explicitly reported as such in the published article or the clinical study report. If none was explicitly reported, we used the outcome chosen for the sample size calculation. If none was identified this way, the study was assessed as not evaluable for analysis of selective outcome reporting. Furthermore, information on the funding source, the number of involved centres, and the study location was extracted.

### Assessing trial registration

All trials reporting a registration number in the published article were deemed as registered. To identify additional registered trials that were unpublished yet or registered trial protocols that were not referenced in the published study, we manually compared the search results of the registries with the electronic database search of published studies. To assess whether trial registration occurred pro- or retrospectively we compared the reported date of first participant’s enrolment (in some registers called “study start”) and the date of trial registration (in some registers called “first posted”). If the date of trial registration was before or at the same date of first participant’s enrolment, the trial was deemed as prospectively registered, otherwise as retrospectively.

### Assessment of the journals policies

We systematically examined in August 2020 (day of assessment) whether the journals having published an eligible trial followed the ICMJE recommendation that clinical trials should be prospectively registered in a trial register. The date of incorporation the ICMJE recommendation in the editorial policy was determined based on the list date reported on the ICMJE website (http://www.icmje.org/journals-following-the-icmje-recommendations/). If the journal was not listed there, the “Instructions for authors” on the journals’ homepage were checked for any information and dates. If there was no information available, we assumed that following the ICMJE recommendation is not a prerequisite for publication in this journal. We compared the list date of adopting the ICMJE recommendation in the journal’s policy with the publication date and the registration status of the trial to assess whether adopting this recommendation increased the number of prospective registrations. We limited this comparisons to the trials published between 2004 and 2017, the time in which trial registration of eligible studies occurred.

### Risk of bias

In the course of the Cochrane review, we assessed the study’s risk of bias using the Cochrane ‘Risk of bias’ assessment tool 1.0. The Cochrane risk of bias domains includes sequence generation, allocation concealment, blinding of participants and personnel, blinding of outcome assessment, incomplete outcome data, selective outcome reporting and ‘other issues’. As detailed in the Cochrane review, the overall risk of bias for each study was assessed by reference to the judgements of the domains ‘sequence generation’, ‘blinding of participant, personnel, and outcome assessors’, and ‘incomplete outcome data’. For the current study, we used and reported in detail the results of the domain ‘selective outcome reporting’.

### Selective outcome reporting

Prospective trial registration was the basis for the risk assessment of selective reporting of outcome results. All non-registered and retrospectively registered trials were assessed as ‘unclear risk’ of selective outcome reporting bias. In all prospectively registered trials, we compared the type and order of outcomes (primary versus secondary) and the times of assessments reported in the trial protocol along with the published outcomes. We used the information on outcomes provided in the latest protocol version. As part of the Cochrane risk of bias assessment, we considered selective outcome reporting as ‘low risk of bias’ if a prospectively registered trial protocol was available and the primary outcome was clearly described and reported in the published trial as pre-specified in the protocol. We judged selective outcome reporting as ‘high risk of bias’ if at least one of the predefined primary outcomes in the registered protocol differed from those in the published study report.

Studies labelled as ‘high risk of bias’ for selective outcome reporting were further investigated for major discrepancies between the registered and published outcomes according to Chan et al. [4] and Mathieu et al. [12]:

1. The registered primary outcome was reported as a secondary outcome in the published article.
2. The registered primary outcome was omitted in the published report.
3. A new primary outcome was introduced in the published article.
4. The published primary outcome was registered as a secondary outcome.
5. The timing of assessment of the registered and published primary outcomes differed.

All prospectively registered trials with major outcome discrepancies (high risk trials) were investigated according to statistical significance of the results [4, 12]. We considered results of the primary outcomes as statistically significant if they reached a significance level of *p* ≤ 0.05 or if they were declared as such by the authors of the published article. If the published study did not provide any information on statistical significance of the primary outcome result, the discrepancy was described as not evaluable. Discrepancies were considered to favour statistically significant results, if:

1. a new statistically significant primary outcome was introduced in the published article, or
2. a non-significant registered primary outcome was defined as non-primary in the published article or omitted.

### Statistical analysis

We calculated median and interquartile ranges (IQR) for continuous variables, and presented absolute and relative frequencies for categorical variables, including overall risk of bias, funding source, and the study location. We used the Pearson’s Chi-squared test when considering single independent variables. *P* ≤ 0.05 was considered statistically significant. Statistical analyses and graphs were produced using RStudio (Integrated Development for R. PBC, Boston, MA).

## Results

A total of 585 RCTs on antiemetic drugs were included and referenced in the Cochrane review [10, 11]. Of these 585 trials, 75 were registered at a clinical study registry (Supplementary File 1 and 2); 58 registered trials have referenced a trial registration number in the journal publication, 11 trials have not referenced any registration number [13–23], and six trials without a full text journal publication have reported results in the trial register or a clinical study report [24–29] (Supplementary File 3). Considering all trial protocols, 83 registrations at 11 different registries were retrieved, taking into account that eight RCTs were registered twice at different registries (Supplementary File 3). The majority of the trials were registered at ClinicalTrials.gov (50/83) followed by the EU Clinical Trials Register (11/83).

Among the 75 registered trials, 36% (27/75) were prospectively and 64% (48/75) retrospectively registered (Supplementary File 3).

**Fig. 1:**
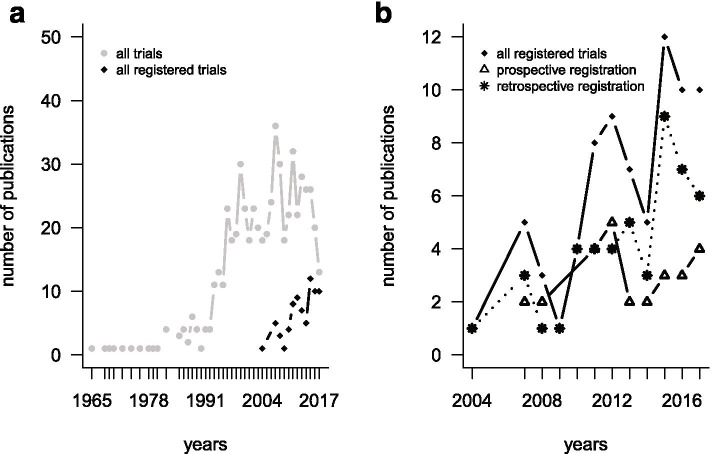
Annual numbers of publications of all included trials (*n* = 585) from 1965 to 2017 (**a**) and of all registered trials (*n* = 75) from 2004 to 2017 (**b**). The first registered trial was published in 2004

Trials of the Cochrane review were published between 1965 and 2017 (Fig. 1a). From 1996 onwards, more than 15 trials have been published annually. The first trial [30] was registered in 2002 and published in 2004. After 2004, the number of registered studies increased over time, with the number of retrospective registrations increasing more than prospective registrations (Fig. 1b). We used the study pool from 2004 to 2017 for further analyses as study authors were less familiar with trial registration before 2004.

The proportion of registered trials among all trials published from 2004 onwards, the time of announcing trial registration as an important prerequisite for publication by the ICMJE, amounts to 22% (75/334) with 8% (27/334) prospective registrations.

Sixty-three percent (47/75) of the registered trials compared to 49% (127/259) of the non-registered trials (2004 to 2017) were published in journals that follow the ICMJE policy of prospective trial registration at the day of assessment (*p* = 0.051; Chi^2^ = 3.802, df = 1). Of the 47 registered trials published in journals that follow the ICMJE recommendation, half of the trials were published before (24/47) and the other half (23/47) after the date the ICMJE recommendation was included in the editorial policies (Supplementary File 4). In contrast, the majority of non-registered trials (120/127) was published before the journals included the ICMJE recommendation. The ICMJE recommendation was included on average two years earlier in the policies of journals publishing registered trials (2014 (IQR 2010 to 2017)) compared to journals publishing non-registered trials (2016 (IQR 2014 to 2018)). Among the registered trials published in journals that follow the ICMJE policy, the proportion of prospective registrations increased from 25% (6/24) prior to the date of adopting the ICMJE recommendation by the journal to 48% (11/23) after adopting (Supplementary File 4).

Selective outcome reporting bias could be evaluated for prospectively registered trials only. Non-registered (259/334) and retrospectively (48/334) registered trials were assessed as unclear risk of selective outcome reporting bias. Of the 27 prospectively registered trials, 41% (11/27) were assessed as ‘low risk’ of selective outcome reporting bias (Supplementary File 3). About one-fifth of the trials (6/27) were assessed as unclear risk of bias due to missing information on outcomes (Supplementary File 3). The remaining 37% of trials (10/27) were assessed as ‘high risk’ of selective outcome reporting bias and were subject to further assessment on the type of major outcome discrepancies between the trial protocol and the publication (Table 1). The 10 ‘high risk’ studies contained a total of 19 major discrepancies (Table 1, Supplementary File 5). About one third (6/19) of all major discrepancies appeared as a switch of the registered primary outcome into a reported secondary outcome in the published articles. In 26% of studies, a new primary outcome was introduced in the published article (5/19) or the timing of assessment of the registered and published primary outcomes differed (5/19). Two trials omitted the registered primary outcome in the published article. Upgrading from a secondary outcome in the protocol to a primary outcome in the published article occurred only once.

**Table 1:** Discrepancies of registered and reported (published) outcomes

Major outcome discrepancies between registry and publication	Number (%)
RCTs with major discrepancies	
all prospectively registered RCTs	27 (100%)
RCTs with major discrepancies between protocol and publication	10 (37.0%)
Type of major discrepancies between registry and publication	
all major discrepancies	19 (100%)
registered primary outcome was reported as a secondary outcome in the published article	6 (31.6%)
new primary outcome was introduced in the published article	5 (26.3%)
timing of assessment of the registered and published primary outcomes differs	5 (26.3%)
registered primary outcome was omitted in the published article	2 (10.5%)
published primary outcome was registered as secondary outcome	1 (5.3%)
Relation of primary outcome discrepancy on statistically significant results in RCTs	
all major discrepancies	19 (100%)
unable to evaluate	4 (21.1%)
evaluable	15 (78.9%)
discrepancy favours statistical significance	12
discrepancy does not favour statistical significance	2
unclear	1
All evaluable published RCTs with discrepancies favouring statistical significance	8 (80%)

Fifteen out of 19 major discrepancies could be evaluated regarding a relation to the statistical significance of the result (Table 1, Supplementary File 3 and 5). Altogether, 12 out of 15 evaluable discrepancies favoured a statistically significant outcome result. With respect to all trials with ‘high risk of bias’ for selective outcome reporting, 80% (8/10) favoured at least one statistically significant result.

We set out to compare registered and non-registered trials in terms of their ‘overall risk of bias’ assessed with the Cochrane Risk of Bias assessment tool, their source of funding, and their geographical location of the study conduct.

**Fig. 2:**
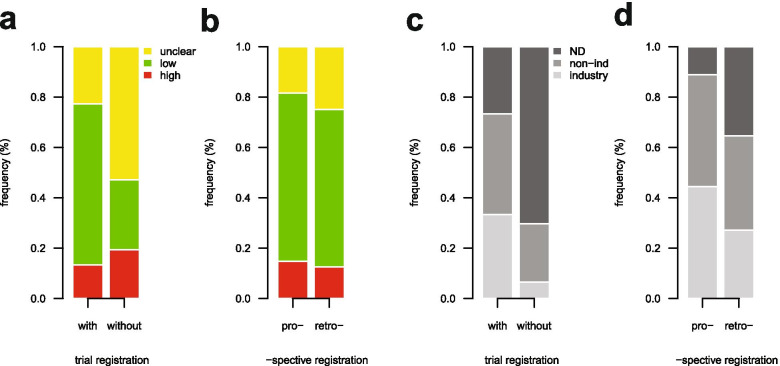
Association between registration status and risk of bias (**a**, **b**), or funding source (**c**, **d**) of included trials. Relative frequency (%) of all published trials (**a** and **c**, *n* = 334) and of all registered trials (**b** and **d**, n = 75) from 2004 to 2017. Risk of bias assessment according to “low”, “unclear”, and “high”. Funding source according to industry, non-industry (non-ind), and not determined (ND)

The proportion of trials rated as overall low, unclear or high risk of bias according to the Cochrane Risk of Bias assessment was different in registered and non-registered trials published between 2004 and 2017 (*p* < 0.001; Chi^2^ = 33.894, df = 2). Registered trials were more often assessed as ‘overall low risk of bias’ compared to non-registered trials (64% vs. 28%) (Fig. 2a). In contrast, non-registered trials were more frequently assessed as ‘overall unclear risk of bias’ (53% vs. 23%). No difference was detectable in the distribution of the ‘overall risk of bias’ assessment between pro- and retrospectively registered trials (*p* = 0.80; Chi^2^ = 0.44, df = 2) (Fig. 2b).

There was a significant difference in the source of funding reported in registered and non-registered trials published between 2004 and 2017 (*p* < 0.001; Chi^2^ = 57.543, df = 2). Registered trials declared their funding source more often compared to non-registered trials (73% vs. 30%) (Fig. 2c). The proportion of studies with industry and non-industry funding among registered trials was 33% (25/75) and 40% (30/75), respectively, and among non-registered trials 7% (17/259) and 23% (60/259), respectively (Fig. 2c). There was no difference detectable in the distribution of the funding source in pro- and retrospectively registered trials (*p* = 0.06; Chi^2^ = 5.60, df = 2) (Fig. 2d).

**Fig. 3:**
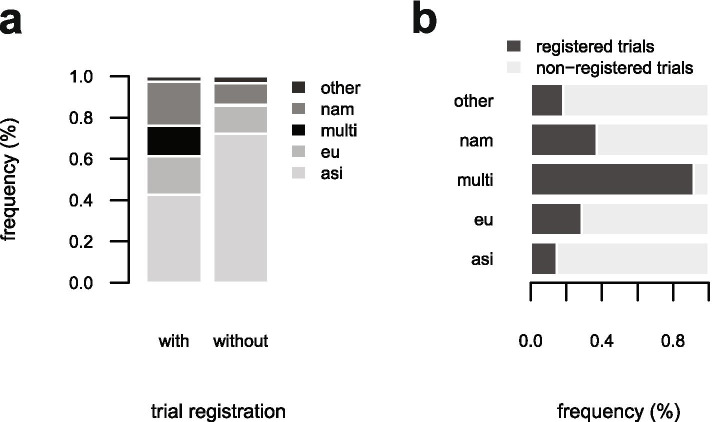
Association between registration status and geographic location of all included trials from 2004 to 2017 (n = 334). Relative frequency (%) plotted for registered and non-registered trials (**a**) and plotted for geographic location (**b**). Asia (asi), Europe (eu), North America (nam), multi-continental locations (multi), and other

About half of the studies included in the Cochrane review were from Asia (298/585), followed by North America (96/585) and Europe (148/585). There was a significant difference in the geographic distribution of registered and non-registered trials published between 2004 and 2017 (*p* < 0.001; Chi^2^ = 47.293, df = 4). Among registered trials the proportion of Asian studies was lower and the proportion of multi-center studies conducted in different continents was higher compared to non-registered trials (Fig. 3a). In total, 92% (11/12) multi-center studies were registered, but only 15% (32/219) Asian studies (Fig. 3b). There was no difference detectable in the geographic distribution of pro- and retrospectively registered trials (*p* = 0.30; Chi2 = 4.84, df = 4). In total, 55% (6/11) multi-continental studies were prospectively registered, but only 25% (8/32) Asian studies.

## Discussion

To evaluate trial registration and selective outcome reporting bias in anaesthesiology trials, we analysed the 585 trials included in the recently published large Cochrane review on drugs for prevention of PONV [10, 11]. In 2004, the first registered trial was published [30]. In the same year, the ICMJE initiative calling for a prospective registration of clinical trials was announced [6]. After the ICMJE announcement 334 included trials were published, but only 22% (75/334) trials were registered. The majority of registered trials were retrospectively registered and only 36% (27/75) of the registrations occurred prospectively. The proportion of prospectively registered studies increased from 25% prior to inclusion of the ICMJE recommendation in the journals’ policies to 48% thereafter. 41% (11/27) of the prospectively registered trials were “free” of selective outcome reporting bias and 37% (10/27) trials showed some evidence of selective outcome reporting with a total of 19 major outcome discrepancies between registered and published trial reports. Among the trials with selective outcome reporting 80% (8/10) favoured reporting of at least one statistically significant result. In general, registered trial compared to non-registered trials were more often of ‘overall low risk of bias’ and reported the source of funding. Most of the multi-center trials were registered, but only a minority of the Asian studies.

Previous studies on trial registration in anaesthesia clinical trials focused mainly on RCTs published in high impact journals of anaesthesiology, pain or critical care during one year or short periods of time [9, 31–33]. Our study provides further insight into the phenomena of trial registration and selective outcome reporting in anaesthesia trials regarding antiemetic drugs. To the best of our knowledge, this is the first study to examine trial registration and selective outcome reporting in a specific field of clinical research in anaesthesia (PONV research) published over time in a broad range of different journals not restricted to the impact factor of the journals. The trial registration rate in our study with 22% (75/334) was lower compared to previous studies on trial registration in anaesthesia trials ranging from 35 to 78% [9, 31–33]. On the one hand, the discrepancy might be due to different observation periods. Our study dates back to 2004 a time period where the recommendation of the ICMJE had not yet gained much awareness. Viergever et al. showed in general that the global number of registered clinical studies increased fivefold between 2004 and 2013 [34]. On the other hand, we did not restrict our study pool to studies published in high impact journals compared to the other studies [9, 31–33]. High impact journals do more frequently require trial registration than low impact journals [35]. In our study, we showed that registered studies were published more frequently in journals that follow the ICMJE policy.

We found that only 8% (27/334) of trials published between 2004 and 2017 were prospectively registered. This finding is comparable to Jones et al. that 14% of the 860 RCTs published in the top 6 general anaesthesiology journals in 2007, 2010, 2013, and 2015 were prospectively registered [9]. Retrospective registration is a major problem, because there is no guarantee that trials have not been registered in favour of a certain result [34]. The frequency of trial registration at all (pro- and retrospective) in studies published in journals following the ICMJE policy did not differ between the time before and after the date of adopting the ICMJE policy. However, we found that the proportion of prospective registrations increased after the endorsement of the ICMJE policy by the journals.

Prospective registration per se does not prevent selective outcome reporting. Only 41% (11/27) trials were free of selective outcome reporting bias and 37% (10/27) were at high risk. Among the leading forms of selective outcome reporting in our study were a change from primary to secondary outcomes, introduction of new primary outcomes, and different outcome assessment times. The influence of these discrepancies could be assessed in two-third of them and in these statistically significant results were favoured in 80% (8/10) of trials. This finding is in line with results from Mathieu et al. who found that 83% of RCTs with outcome discrepancies published in 2008 in 10 general medical high impact journals favoured statistically significant results [12].

Trial registration can be seen as a quality criteria of clinical studies. We found that registered trials were more frequently assessed as ‘overall low risk of bias’ with the Cochrane Risk of Bias tool and reported their funding source. Therefore, trial registration is a reliable quality criteria for RCTs in the context of our study. Interestingly, Asian studies that make the largest proportion of trials in the Cochrane review tend to be less frequently registered with only 15% (32/219) of all Asian studies compared to studies conducted in other areas of the world, especially Europe and North America. When the studies were carried out as multi-centre studies in different centres over the world, they were registered mostly.

Our study has limitations. The current study was not prospectively registered. The primary aim of our group was to conduct a systematic Cochrane review for PONV prophylaxis. It was only during our work that we came across the issue of missing trial registration and selective outcome reporting in the field of PONV research. We were not aware of the extent of this problem before the start of the review and wanted to share our findings with the scientific community. Therefore, we could have registered only a protocol for this study retrospectively. However, retrospective registration would be in contradiction to the message of our paper. As part of the previously published Cochrane systematic review on antiemetic drugs, we performed a comprehensive literature search which was also the basis for this study. Several studies had duplicate publications in different journals and were listed in trial registries by different first authors; this complicated the process of data synthesis. By making references and dataset freely available [10], we welcome perusal by outside researchers to identify mistakes in our dataset, our analysis or our interpretation. We did not send requests to trial authors either not publishing a trial registration number in the publication asking for trial registration or not providing sufficient details in the protocol to assess selective outcome reporting. Thus, the proportion of registered trials could be underestimated and the proportion of selective outcome reporting in prospectively registered trials could be slightly different. We defined prospectively registered trials as those in which the date of trial registration was before or at the same date of first participant’s enrolment. Thus, we may have underestimated the number of prospectively registered trials by definition. However, the chosen approach deemed sensible to have clearly defined time-points. We assessed selective outcome reporting and trial registration for trials published from 2004 onwards which is the same year that ICMJE introduced trial registration as a necessity for good clinical practice and the first of our included published studies registered the trial. Due to the lag from trial initiation to publication of results, trialists publishing shortly after the ICMJE’s announcement certainly would not be able to offer a prospective protocol. Therefore, an increase in prospective trial registration is expectedly delayed. Additionally, it has to be kept in mind that this study only considers trials investigating antiemetic drugs for PONV prophylaxis and our findings cannot be extrapolated to other fields in anaesthesiology research.

Finally, only 3% (11/334) of the investigated trials were deemed as prospectively and completely registered and free of selective outcome reporting. This is still an alarming sign of inadequacy in clinical research of PONV despite more than ten years of international recommendations on prospective trial registration. However, PONV research is not an outsider in this field since other specialities, e.g. psychotherapy, reported similar numbers with only 5% of RCTs that were free of selective outcome reporting [36]. Therefore, study’s authors should became aware of that a complete, transparent, and prospective trial registration and publication according to the specifications set out in the protocol is seen as an important quality criteria that increases the confidence of the study findings.

## Conclusions

In 2017, 13 years after the ICMJE declared prospective protocol registration a necessity for reliable clinical studies, the frequency and quality of trial registration in the field of PONV research is very poor. Only one fifth of the clinical trials published in 2004 or later and included in the recently published Cochrane review referenced a registered trial protocol of which almost two third were registered retrospectively. In the end, of the prospectively registered trials less than 50% were free of selective outcome reporting bias. This is an alarming deficit. We also showed that registered trials in general were more frequently judged as overall low risk of bias regarding the Cochrane Risk of Bias assessment, suggesting trial registration as a quality criterion for RCTs in PONV clinical research. Selective outcome reporting reduces trustworthiness in findings of clinical trials. Investigators and clinicians should be aware that only following a properly registered protocol and transparently reporting of predefined outcomes, regardless of the direction and significance of the result, will ultimately strengthen the body of evidence in the field of PONV research in the future.

## Supplementary Information

**Additional file 1.** PRISMA study flow diagram.

**Additional file 2.** References to included registered studies.

**Additional file 3.** Studies with registered study protocols.

**Additional file 4.** List of journals publishing the registered trials (*n* = 75), with date of adopting the ICMJE policy.

**Additional file 5.** Major discrepancies of prospectively registered studies with high risk of selective outcome reporting (SOR).

## Data Availability

The datasets used and/or analysed during the current study are available from the corresponding author on reasonable request.

## Abbreviations

PONV: Postoperative Nausea and Vomiting
ICMJE: International Committee of Medical Journal Editors
RCT: Randomized Clinical Trials
WHO: World Health Organization
CENTRAL: Cochrane Central Register of Controlled Trials
ICTRP: International Clinical Trials Registry Platform
CINAHL: Cumulative Index to Nursing and Allied Health Literature
IQR: Interquartile Ranges
EU: European Union
