# Supplementary material for: Trial registration and selective outcome reporting in 585 clinical trials investigating drugs for prevention of postoperative nausea and vomiting
Source: BMC Anesthesiol. 2021 Oct 19;21:249. doi: 10.1186/s12871-021-01464-w (PMC8524993; doi:10.1186/s12871-021-01464-w)
Supplement: Supplementary file 1 — Additional file 1. PRISMA study flow diagram. [file 12871_2021_1464_MOESM1_ESM.pdf]

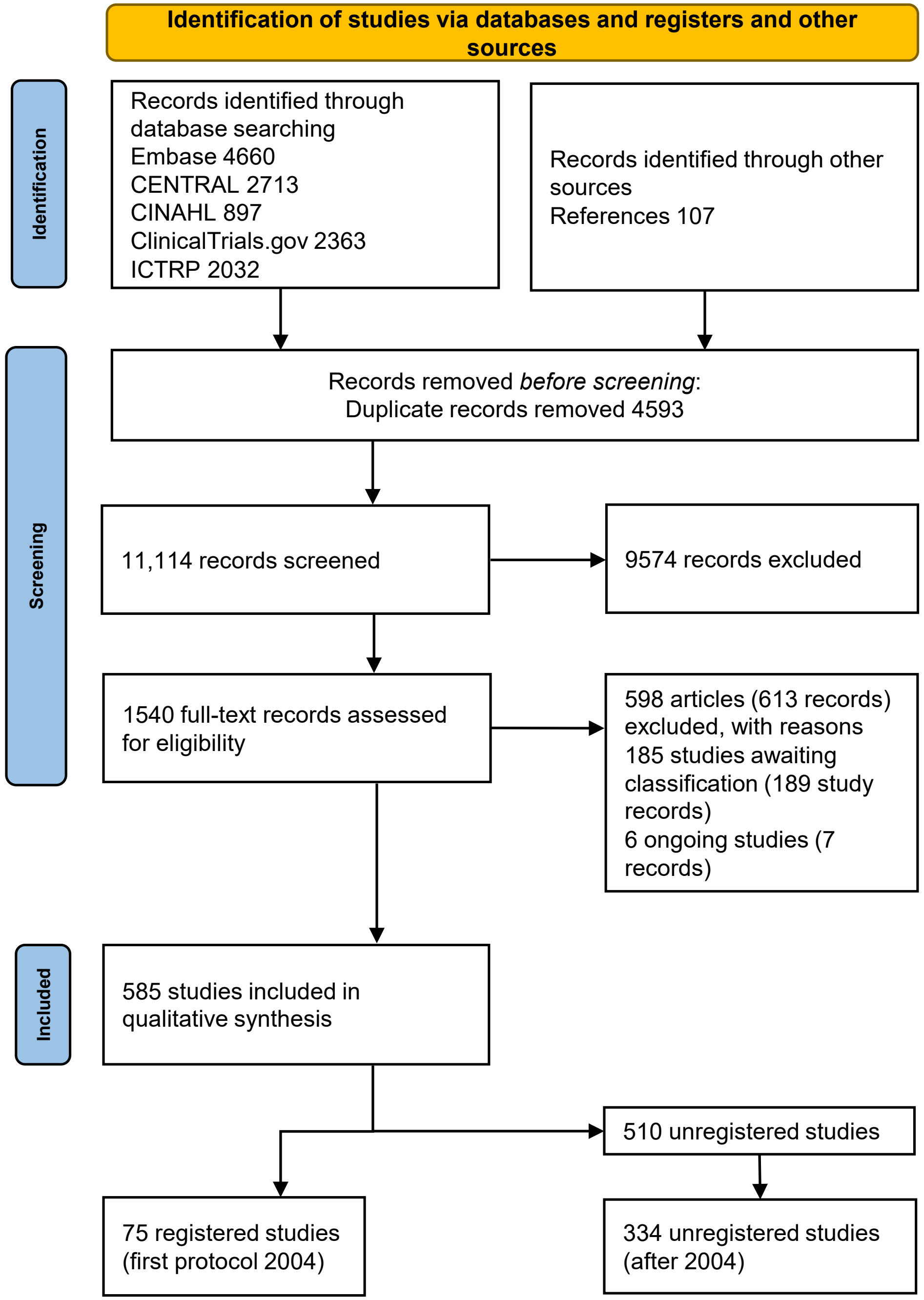

*Consider, if feasible to do so, reporting the number of records identified from each database or register searched (rather than the total number across all databases/registers).

**If automation tools were used, indicate how many records were excluded by a human and how many were excluded by automation tools.

From: Page MJ, McKenzie JE, Bossuyt PM, Boutron I, Hoffmann TC, Mulrow CD, et al. The PRISMA 2020 statement: an updated guideline for reporting systematic reviews. BMJ 2021;372:n71. doi: 10.1136/bmj.n71. For more information, visit: http://www.prisma-statement.org/
