# Supplementary material for: Trial registration and selective outcome reporting in 585 clinical trials investigating drugs for prevention of postoperative nausea and vomiting
Source: BMC Anesthesiol. 2021 Oct 19;21:249. doi: 10.1186/s12871-021-01464-w (PMC8524993; doi:10.1186/s12871-021-01464-w)
Supplement: Supplementary file 2 — Additional file 2. References to included registered studies. [file 12871_2021_1464_MOESM2_ESM.pdf]

Supplementary File 2: References to included registered studies [1-75]

1. Agarkar S, Chatterjee AS: Comparison of ramosetron with ondansetron for the prevention of post-operative nausea and vomiting in high-risk patients. Indian Journal of Anaesthesia 2015; 59: 222-227

2. Alghanem SM, Massad IM, Rashed EM, Abu-Ali HM, Daradkeh SS: Optimization of anesthesia antiemetic measures versus combination therapy using dexamethasone or ondansetron for the prevention of postoperative nausea and vomiting. Surgical Endoscopy 2010; 24: 353-358

3. Altorjay A, Melson T, Chinachoit T, Kett A, Aqua K, Levin J, et al: Casopitant and ondansetron for postoperative nausea and vomiting prevention in women at high risk for emesis: a phase 3 study. Archives of Surgery (Chicago, Ill. : 1960) 2011; 146: 201-206

4. Apfel CC, Korttila K, Abdalla M, Kerger H, Turan A, Vedder I, et al: A factorial trial of six interventions for the prevention of postoperative nausea and vomiting. New England Journal of Medicine 2004; 350: 2441-2451

5. Areeruk P, Ittichaikulthol W, Termpornlert S, Pravitharangul T, Nganlasom J, Charoensap C, et al: The effect of a single dose dexamethasone on postoperative pain in patients undergoing gynecological laparotomy surgery. Chotmaihet Thangphaet [Journal of the Medical Association of Thailand] 2016; 99: 1239-1244

6. Bang SR, Kim HJ, Ahn EJ, Choi HR, Kim KW, Ko MJ, et al: Effect of total intravenous anesthesia and prophylactic 5-HT3 receptor antagonist on postoperative nausea and vomiting after gynecologic laparoscopic surgery: a prospective, randomized controlled study. Rawal Medical Journal 2017; 42: 73-77

7. Bang YS, Kim YU, Oh D, Shin EY, Park SK: A randomized, double-blind trial evaluating the efficacy of palonosetron with total intravenous anesthesia using propofol and remifentanil for the prevention of postoperative nausea and vomiting after gynecologic surgery. Journal of Anesthesia 2016; 30: 935-940

8. Bataille A, Letourneulx JF, Charmeau A, Lemedioni P, Léger P, Chazot T, et al: Impact of a prophylactic combination of dexamethasone-ondansetron on postoperative nausea and vomiting in obese adult patients undergoing laparoscopic sleeve gastrectomy

during closed-loop propofol-remifentanil anaesthesia: A randomised double-blind placebo-controlled study. European Journal of Anaesthesiology 2016; 33: 898-905

9. Benevides ML, Oliveira SS, de Aguilar-Nascimento JE: The combination of haloperidol, dexamethasone, and ondansetron for prevention of postoperative nausea and vomiting in laparoscopic sleeve gastrectomy: a randomized double-blind trial. Obesity Surgery 2013; 23: 1389-1396

10. Bergese SD, Puente EG, Antor MA, Viloria AL, Yildiz V, Kumar NA, et al: A prospective, randomized, double-blinded, double-dummy pilot study to assess the preemptive effect of triple therapy with aprepitant, dexamethasone, and promethazine versus ondansetron, dexamethasone and promethazine on reducing the incidence of postoperative nausea and vomiting experienced by patients undergoing craniotomy under general anesthesia. Frontiers in Medicine 2016; 3: 29-29

11. Bjerregaard LS, Jensen PF, Bigler DR, Petersen RH, Møller-Sørensen H, Gefke K, et al: High-dose methylprednisolone in video-assisted thoracoscopic surgery lobectomy: a randomized controlled trial. European Journal of Cardio Thoracic Surgery 2018; 53: 209-215

12. Blitz JD, Haile M, Kline R, Franco L, Didehvar S, Pachter HL, et al: A randomized double blind study to evaluate efficacy of palonosetron with dexamethasone versus palonosetron alone for prevention of postoperative and postdischarge nausea and vomiting in subjects undergoing laparoscopic surgeries with high emetogenic risk. American Journal of Therapeutics 2012; 19: 324-329

13. Choi EK, Kim DG, Jeon Y: Comparison of the prophylactic antiemetic efficacy of aprepitant plus palonosetron versus aprepitant plus ramosetron in patients at high risk for postoperative nausea and vomiting after laparoscopic cholecystectomy: a prospective randomized-controlled trial. Surgical Laparoscopy, Endoscopy and Percutaneous Techniques 2016; 26: 354-357

14. Chun HR, Jeon IS, Park SY, Lee SJ, Kang SH, Kim SI: Efficacy of palonosetron for the prevention of postoperative nausea and vomiting: a randomized, double-blinded, placebo-controlled trial. British Journal of Anaesthesia 2014; 112: 485-490

15. Corcoran T, Paech M, Law D, Muchatuta NA, French M, Ho KM: Intraoperative dexamethasone alters immune cell populations in patients undergoing elective laparoscopic gynaecological surgery. British Journal of Anaesthesia 2017; 119: 221-230

16. Cortes-Flores AO, Jimenez-Tornero J, Morgan-Villela G, Delgado-Gomez M, Zuloaga-Fernandez Del Valle CJ, Garcia-Renteria J, et al: Effects of preoperative dexamethasone on postoperative pain, nausea, vomiting and respiratory function in women undergoing conservative breast surgery for cancer: results of a controlled clinical trial. European Journal of Cancer Care 2018; 27: e12686-e12686

17. de Oliveira Gs Jr, Ahmad S, Fitzgerald PC, Marcus RJ, Altman CS, Panjwani AS, et al: Dose ranging study on the effect of preoperative dexamethasone on postoperative quality of recovery and opioid consumption after ambulatory gynaecological surgery. British Journal of Anaesthesia 2011; 107: 362-371

18. de Oliveira Gs Jr, Bialek J, Marcus RJ, McCarthy R: Dose-ranging effect of systemic diphenhydramine on postoperative quality of recovery after ambulatory laparoscopic surgery: a randomized, placebo-controlled, double-blinded, clinical trial. Journal of Clinical Anesthesia 2016; 34: 46-52

19. Dewinter G, Teunkens A, Vermeulen K, Devroe S, Van Hemelrijck J, Meuleman C, et al: Alizapride and ondansetron for the prevention of postoperative nausea and vomiting in patients undergoing laparoscopic gynaecological surgery: a double-blind, randomised, placebo-controlled noninferiority study. European Journal of Anaesthesiology 2016; 33: 96-103

20. Diemunsch P, Gan TJ, Philip BK, Girao MJ, Eberhart L, Irwin MG, Aprepitant-Ponv Protocol 091 International Study Group, et al.: Single-dose aprepitant vs ondansetron for the prevention of postoperative nausea and vomiting: a randomized, double-blind phase III trial in patients undergoing open abdominal surgery. British Journal of Anaesthesia 2007; 99: 202-211

21. Eidi M, Kolahdouzan K, Hosseinzadeh H, Tabaqi R: A comparison of preoperative ondansetron and dexamethasone in the prevention of post-tympanoplasty nausea and vomiting. Iranian Journal of Medical Sciences 2012; 37: 166-172

22. Fahlenkamp AV, Stoppe C, Cremer J, Biener IA, Peters D, Leuchter R, et al: Nausea and vomiting following balanced xenon anesthesia compared to sevoflurane: A Post-Hoc explorative analysis of a randomized controlled trial. PLOS One 2016; 11: e0153807-e0153807

23. Feroci F, Rettori M, Borrelli A, Lenzi E, Ottaviano A, Scatizzi M: Dexamethasone prophylaxis before thyroidectomy to reduce postoperative nausea, pain, and vocal dysfunction: a randomized clinical controlled trial. Head and Neck 2011; 33: 840-846

24. Gan TJ, Apfel CC, Kovac A, Philip BK, Singla N, Minkowitz H, Aprepitant-Ponv Study Group, et al.: A randomized, double-blind comparison of the NK1 antagonist, aprepitant, versus ondansetron for the prevention of postoperative nausea and vomiting. Anesthesia and Analgesia 2007; 104: 1082-1089

25. Gan TJ, Gu J, Singla N, Chung F, Pearman MH, Bergese SD, Rolapitant Investigation Group, et al.: Rolapitant for the prevention of postoperative nausea and vomiting: a prospective, double-blinded, placebo-controlled randomized trial. Anesthesia and Analgesia 2011; 112: 804-812

26. Gan TJ, Kranke P, Minkowitz HS, Bergese SD, Motsch J, Eberhart L, et al: Intravenous amisulpride for the prevention of postoperative nausea and vomiting: two concurrent, randomized, double-blind, placebo-controlled trials. Anesthesiology 2017; 126: 268-275

27. Gómez-Hernández J, Orozco-Alatorre AL, Domínguez-Contreras M, Oceguera-Villanueva A, Gómez-Romo S, Alvarez Villaseñor AS, et al: Preoperative dexamethasone reduces postoperative pain, nausea and vomiting following mastectomy for breast cancer. BMC Cancer 2010; 10: 692-692

28. Green MS, Green P, Malayaman SN, Hepler M, Neubert LJ, Horrow JC: Randomized, double-blind comparison of oral aprepitant alone compared with aprepitant and transdermal scopolamine for prevention of postoperative nausea and vomiting. British Journal of Anaesthesia 2012; 109: 716-722

29. Habib AS, Keifer JC, Borel CO, White WD, Gan TJ: A comparison of the combination of aprepitant and dexamethasone versus the combination of ondansetron and dexamethasone for the prevention of postoperative nausea and vomiting in patients undergoing craniotomy. Anesthesia and Analgesia 2011; 112: 813-818

30. Hemmati HR, Ghorbani R, Hossein-Zadeh B, Ebrahim-Zadeh H, Shakeri S: The effect of single dose of dexamethasone on postoperative nausea and vomiting in patients undergoing laparoscopic cholecystectomy. Journal of Babol University of Medical Sciences 2014; 16: 15-21

31. Hessami MA, Yari M: Granisetron versus dexamethasone in prophylaxis of nausea and vomiting after laparoscopic cholecystectomy. Anesthesiology and Pain Medicine 2012; 2: 81-84

32. Hu X, Tan F, Gong L: Higher dose of palonosetron versus lower dose of palonosetron plus droperidol to prevent postoperative nausea and vomiting after eye enucleation and orbital hydroxyapatite implant surgery: a randomized, double-blind trial. Drug design, Development and Therapy 2017; 11: 1465-1472

33. Joo J, Park S, Park HJ, Shin SY: Ramosetron versus ondansetron for postoperative nausea and vomiting in strabismus surgery patients. BMC Anesthesiology 2016; 16: 41-41

34. Joo J, Park YG, Baek J, Moon YE: Haloperidol dose combined with dexamethasone for PONV prophylaxis in high-risk patients undergoing gynecological laparoscopic surgery: a prospective, randomized, double-blind, dose-response and placebo-controlled study. BMC Anesthesiology 2015; 15: 99-99

35. Jung WS, Kim YB, Park HY, Choi WJ, Yang HS: Oral administration of aprepitant to prevent postoperative nausea in highly susceptible patients after gynecological laparoscopy. Journal of Anesthesia 2013; 27: 396-401

36. Kakuta N, Kume K, Hamaguchi E, Tsutsumi R, Mita N, Tanaka K, et al: The effects of intravenous fosaprepitant and ondansetron in the prevention of postoperative nausea and vomiting in patients who underwent lower limb surgery: a prospective, randomized, double-blind study. Journal of Anesthesia 2015; 29: 836-841

37. Kakuta N, Tsutsumi YM, Horikawa YT, Kawano H, Kinoshita M, Tanaka K, et al: Neurokinin-1 receptor antagonism, aprepitant, effectively diminishes post-operative nausea and vomiting while increasing analgesic tolerance in laparoscopic gynecological procedures. Journal of Medical Investigation 2011; 58: 246-251

38. Kim SH, Hong JY, Kim WO, Kil HK, Karm MH, Hwang JH: Palonosetron has superior prophylactic antiemetic efficacy compared with ondansetron or ramosetron in high-risk patients undergoing laparoscopic surgery: a prospective, randomized, double-blinded study. Korean Journal of Anesthesiology 2013; 64: 517-523

39. Kim SH, Oh CS, Lee SJ: Efficacy of palonosetron and ramosetron on postoperative nausea and vomiting related to intravenous patient-controlled analgesia with opioids after gynecological laparoscopic surgery (double-blinded prospective randomized controlled trial). Journal of Anesthesia 2015; 29: 585-592

40. Kim WJ, Kang H, Shin HY, Baek CW, Jung YH, Woo YC, et al: Ramosetron, midazolam, and combination of ramosetron and midazolam for prevention of postoperative

nausea and vomiting: a prospective, randomized, double-blind study. Journal of International Medical Research 2013; 41: 1203-1213

41. Kizilcik N, Bilgen S, Menda F, Türe H, Aydın B, Kaspar EC, et al: Comparison of dexamethasone-dimenhydrinate and dexamethasone-ondansetron in prevention of nausea and vomiting in postoperative patients. Aesthetic Plastic Surgery 2017; 41: 204-210

42. Ko-Iam W, Sandhu T, Paiboonworachat S, Pongchairerks P, Junrungsee S, Chotirosniramit A, et al: Metoclopramide, versus its combination with dexamethasone in the prevention of postoperative nausea and vomiting after laparoscopic cholecystectomy: a double-blind randomized controlled trial. Journal of the Medical Association of Thailand 2015; 98: 265-272

43. Koyuncu O, Leung S, You J, Oksar M, Turhanoglu S, Akkurt C, et al: The effect of ondansetron on analgesic efficacy of acetaminophen after hysterectomy: a randomized double blinded placebo controlled trial. Journal of Clinical Anesthesia 2017; 40: 78-83

44. Kranke P, Eberhart L, Motsch J, Chassard D, Wallenborn J, Diemunsch P, et al: I.V. APD421 (amisulpride) prevents postoperative nausea and vomiting: a randomized, double-blind, placebo-controlled, multicentre trial. British Journal of Anaesthesia 2013; 111: 938-945

45. Kranke P, Röhm KD, Diemunsch P, Gan TJ, Apfel CC, Eberhart L, et al: Intravenous buspirone for the prevention of postoperative nausea and vomiting. European Journal of Clinical Pharmacology 2012; 68: 1465-1472

46. Kurz A, Fleischmann E, Sessler DI, Buggy DJ, Apfel C, Akça O: Effects of supplemental oxygen and dexamethasone on surgical site infection: a factorial randomized trial. British Journal of Anaesthesia 2015; 115: 434-443

47. Lee WS, Lee KB, Lim S, Chang YG: Comparison of palonosetron, granisetron, and ramosetron for the prevention of postoperative nausea and vomiting after laparoscopic gynecologic surgery: a prospective randomized trial. BMC Anesthesiology 2015; 15: 121-121

48. Mathiesen O, Jørgensen DG, Hilsted KL, Trolle W, Stjernholm P, Christiansen H, et al: Pregabalin and dexamethasone improves post-operative pain treatment after tonsillectomy. Acta Anaesthesiologica Scandinavica 2011; 55: 297-305

49. Mathiesen O, Rasmussen ML, Dierking G, Lech K, Hilsted KL, Fomsgaard JS, et al: Pregabalin and dexamethasone in combination with paracetamol for postoperative pain control after abdominal hysterectomy. A randomized clinical trial. Acta Anaesthesiologica Scandinavica 2009; 53: 227-235

50. Murphy GS, Szokol JW, Avram MJ, Greenberg SB, Shear T, Vender JS, et al: The effect of single low-dose dexamethasone on blood glucose concentrations in the perioperative period: a randomized, placebo-controlled investigation in gynecologic surgical patients. Anesthesia and Analgesia 2014; 118: 1204-1212

51. NCT00888329: Aprepitant for Prevention of Postoperative Nausea and Vomiting in Elective Hysterectomy, clinicaltrials.gov/ct2/show/NCT00888329, 2009

52. Nielsen RV, Siegel H, Fomsgaard JS, Andersen JD, Martusevicius R, Mathiesen O, et al: Preoperative dexamethasone reduces acute but not sustained pain after lumbar disk surgery: a randomized, blinded, placebo-controlled trial. Pain 2015; 156: 2538-2544

53. NKO101287: The purpose of this study is to determine the effectiveness of this medication in preventing nausea and vomiting in female patients at risk for post-operative nausea and vomiting (PONV), www.gsk-studyregister.com/study?uniqueStudyId=NKO101287, 2018

54. NKT102245: The primary purpose of this study is to determine an effective dose of this NK-1 anti-emetic medication to prevent nausea and vomiting in females after surgery, www.gsk-studyregister.com/study?uniqueStudyId=NKT102245, 2018

55. NKT102260: This study is looking at a range of doses of this NK-1 receptor antagonist drug, for both safety and effectiveness in prevention PONV, www.gsk-studyregister.com/study?uniqueStudyId=NKT102260, 2018

56. NKT102552: This study is being conducted to see if adding GW679769 (casopitant) to ZOFRAN will significantly decrease the number of patients who experience nausea and vomiting after surgery, www.gsk-studyregister.com/study?uniqueStudyId=NKT102552, 2018

57. PALO-04-07: A Randomized, Double-blind, Multicenter, Parallel Group, Balanced, Stratified Phase 3 Study to Evaluate the Efficacy and the Safety of Single IV Doses of Palonosetron 0.025 mg, 0.050 mg, and 0.075 mg versus Placebo to Prevent Postoperative Nausea and Vomiting Following Elective Gynecologic or Breast Surgery, www.clinicaltrialsregister.eu/ctr-search/search?query=2005-000298-23, 2005

58. Park SK, Cho EJ, Kang SH, Lee YJ, Kim DA: A randomized, double-blind study to evaluate the efficacy of ramosetron and palonosetron for prevention of postoperative nausea and vomiting after gynecological laparoscopic surgery. Korean Journal of Anesthesiology 2013; 64: 133-137

59. Rettori M, Feroci F, Borrelli A, Ottaviano A, Coppola A, Castagnoli A, et al: A single effective way to reduce post-thyroidectomy discomfort: a clinical trial. Minerva Chirurgica 2011; 66: 197-205

60. Ryoo SH, Yoo JH, Kim MG, Lee KH, Kim SI: The effect of combination treatment using palonosetron and dexamethasone for the prevention of postoperative nausea and vomiting versus dexamethasone alone in women receiving intravenous patient-controlled analgesia. Korean Journal of Anesthesiology 2015; 68: 267-273

61. Ryu JH, Chang JE, Kim HR, Hwang JW, Oh AY, Do SH: Ramosetron vs. ramosetron plus dexamethasone for the prevention of postoperative nausea and vomiting (PONV) after laparoscopic cholecystectomy: prospective, randomized, and double-blind study. International Journal of Surgery 2013; 11: 183-187

62. Sánchez-Rodríguez PE, Fuentes-Orozco C, González-Ojeda A: Effect of dexamethasone on postoperative symptoms in patients undergoing elective laparoscopic cholecystectomy: randomized clinical trial. World Journal of Surgery 2010; 34: 895-900

63. Segelman J, Pettersson H, Svensen C, Divander MB, Barenius B, Segelman J: Analgesic effect of a single dose of betamethasone after ambulatory knee arthroscopy: a randomized controlled trial. Journal of Anesthesia 2016; 30: 803-810

64. Singla NK, Singla SK, Chung F, Kutsogiannis DJ, Blackburn L, Lane SR, et al: Phase II study to evaluate the safety and efficacy of the oral neurokinin-1 receptor antagonist casopitant (GW679769) administered with ondansetron for the prevention of postoperative and postdischarge nausea and vomiting in high-risk patients. Anesthesiology 2010; 113: 74-82

65. Sinha AC, Singh PM, Williams NW, Ochroch EA, Goudra BG: Aprepitant's prophylactic efficacy in decreasing postoperative nausea and vomiting in morbidly obese patients undergoing bariatric surgery. Obesity Surgery 2014; 24: 225-231

66. Soga T, Kume K, Kakuta N, Hamaguchi E, Tsutsumi R, Kawanishi R, et al: Fosaprepitant versus ondansetron for the prevention of postoperative nausea and vomiting in patients who undergo gynecologic abdominal surgery with patient-controlled epidural analgesia: a prospective, randomized, double-blind study. Journal of Anesthesia 2015; 29: 696-701

67. Song JW, Shim JK, Choi SH, Soh S, Jang J, Kwak YL: Comparison of Ramosetron and Palonosetron for Preventing Nausea and Vomiting after Spinal Surgery: Association With ABCB1 Polymorphisms. Journal of Neurosurgical Anesthesiology 2017; 29: 406-414

68. Tarantino I, Warschkow R, Beutner U, Kolb W, Luthi A, Luthi C, Schmied BM, Clerici T: Efficacy of a single preoperative dexamethasone dose to prevent nausea and vomiting after thyroidectomy (the tPONV Study): a randomized, double-blind, placebo-controlled clinical trial. Annals of surgery 2015; 262: 934-940

69. Tolver MA, Strandfelt P, Bryld EB, Rosenberg J, Bisgaard T: Randomized clinical trial of dexamethasone versus placebo in laparoscopic inguinal hernia repair. British Journal of Surgery 2012; 99: 1374-1380

70. Tsutsumi YM, Kakuta N, Soga T, Kume K, Hamaguchi E, Tsutsumi R, Tanaka K: The effects of intravenous fosaprepitant and ondansetron for the prevention of postoperative nausea and vomiting in neurosurgery patients: a prospective, randomized, double-blinded study. Biomed Research International 2014; 2014: 307025-307025

71. Vallejo MC, Phelps AL, Ibinson JW, Barnes LR, Milord PJ, Romeo RC, et al: Aprepitant plus ondansetron compared with ondansetron alone in reducing postoperative nausea and vomiting in ambulatory patients undergoing plastic surgery. Plastic and Reconstructive Surgery 2012; 129: 519-526

72. Worni M, Schudel HH, Seifert E, Inglin R, Hagemann M, Vorburger SA, et al: Randomized controlled trial on single dose steroid before thyroidectomy for benign disease to improve postoperative nausea, pain, and vocal function. Annals of Surgery 2008; 248: 1060-1066

73. Yang C, Jung SM, Bae YK, Park SJ: The effect of ketorolac and dexamethasone on the incidence of sore throat in women after thyroidectomy: a prospective double-blinded randomized trial. Korean Journal of Anesthesiology 2017; 70: 64-71

74. Yang XY, Xiao J, Chen YH, Wang ZT, Wang HL, He DH, et al: Dexamethasone alone vs in combination with transcutaneous electrical acupoint stimulation or tropisetron for prevention of postoperative nausea and vomiting in gynaecological patients undergoing laparoscopic surgery. British Journal of Anaesthesia 2015; 115: 883-889

75. Zhou H, Xu H, Zhang J, Wang W, Wang Y, Hu Z: Combination of dexamethasone and tropisetron before thyroidectomy to alleviate postoperative nausea, vomiting, and pain: randomized controlled trial. World Journal of Surgery 2012; 36: 1217-1224
