# Supplementary material for: Trial registration and selective outcome reporting in 585 clinical trials investigating drugs for prevention of postoperative nausea and vomiting
Source: BMC Anesthesiol. 2021 Oct 19;21:249. doi: 10.1186/s12871-021-01464-w (PMC8524993; doi:10.1186/s12871-021-01464-w)
Supplement: Supplementary file 3 — Additional file 3. Studies with registered study protocols. [file 12871_2021_1464_MOESM3_ESM.pdf]

Supplementary File 3: Studies with registered study protocols

Study-ID	Protocol number	Pro-/retrospective registration	Risk of selective outcome reporting bias (SOR)	SOR: Number of major discrepancies
Agarkar-2015	CTRI/2013/02/003357	retrospective	unclear	-
Alghanem-2010	NCT00825071	retrospective	unclear	-
Altorjay-2011	(Pro) EUCTR2005-005856-42-GB, (Retro) NCT00326248	pro and retrospective	low	-
Apfel-2004	Controlled Clinical Trials 24 (2003), 736-751	retrospective	unclear	-
Areeruk-2016	TCTR20151116001	retrospective	unclear	-
Bang-2016	NCT01478165	retrospective	unclear	-
Bang-2017	KCT0001930	retrospective	unclear	-
Bataille-2016	NCT01876290	retrospective	unclear	-
Benevides-2013	RBR-4bxdhb	retrospective	unclear	-
Bergese-2016	NCT01474915	retrospective	unclear	-
Bjerregaard-2018	EUCTR2012-004451-3	prospective	low	-
Blitz-2012	NCT00952133	retrospective	unclear	-
Choi-2016	NCT02597907	retrospective	unclear	-
Chun-2014	KCT0000537	retrospective	unclear	-
Corcoran-2017	ACTRN12608000340336	prospective	unclear (either protocol or study article provides no information about the "time assessment")	-
Cortes-Flores-2018	NCT02305173	retrospective	unclear	-
DeOliveira-2011	NCT01052038	prospective	high	4
DeOliveira-2016	NCT01451762	retrospective	unclear	-
Dewinter-2016	EUCTR2008-004789-20	prospective	unclear (either protocol or study article provides no information about the "time assessment")	-
Diemunsch-2007	NCT00090246	retrospective	unclear	-
Eidi-2012	IRCT201106154005N4	retrospective	unclear	-
Fahlenkamp-2016	EUCTR2008-004132-20, NCT00793663	prospective	high	2
Feroci-2011	NCT00888303	retrospective	unclear	-
Gan-2007	NCT00090155	retrospective	unclear	-
Gan-2011	NCT00539721	prospective	low	-
Gan-2017	NCT01991821, NCT01991860 (US Phase)	retrospective	unclear	-
Gomez-Hernandez-2010	NCT01116713	retrospective	unclear	-
Green-2012	NCT00717054,	prospective	high	2

	NCT00659737			
Habib-2011	NCT00734929	retrospective	unclear	-
Hemmati-2014	IRCT2014062118168N1	retrospective	unclear	-
Hessami-2012	IRCT138903033939N1	retrospective	unclear	-
Hu-2017	ChiCTR-IPR-15005852	prospective	high	2
Joo-2015	NCT01639599	retrospective	unclear	-
Joo-2016	KCT0000688	retrospective	unclear	-
Jung-2013	NCT01440673	retrospective	unclear	-
Kakuta-2011	JPRN-UMIN000003850	prospective	unclear (the study does not declare its primary outcome)	-
Kakuta-2015	UMIN000012999	prospective	unclear (either protocol or study article provides no information about the "time assessment")	-
Kim-2015	KCT0000183	unclear due to contradictory registration data (assessed as retrospective)	-	-
Kim-SH-2013	NCT01169805	prospective	high	2
Kim-WJ-2013	ACTRN12612000757819	retrospective	unclear	-
Kizilcik-2017	NCT02382146	retrospective	unclear	-
Ko-iam-2015	TCTR20140128001	retrospective	unclear	-
Koyuncu-2017	NCT02296333	retrospective	unclear	-
Kranke-2012	NCT00895830	prospective	low	-
Kranke-2013	NCT01510704, EUCTR2011-004267-71	prospective	low	-
Kurz-2015	NCT00273377	retrospective	unclear	-
Lee-WS-2015	NCT01752374	retrospective	unclear	-
Mathiesen-2009	NCT00209495	retrospective	unclear	-
Mathiesen-2011	NCT00378547	retrospective	unclear	-
Murphy-2014	NCT01545700	retrospective	unclear	-
NCT00888329	NCT00888329	retrospective	unclear	-
Nielsen-2015	NCT01953978	retrospective	unclear	-
NKO101287	(Pro) EUCTR2004-001021-22, (Retro) NCT00600990	pro and retrospective	low	-
NKT102245	(Pro) NCT00274690, (Retro) EUCTR2004-000369-37	pro and retrospective	low	-
NKT102260	EUCTR2004-000370-31, NCT00108095	retrospective	unclear	-
NKT102552	EUCTR2005-005855-16-BE	prospective	low	-
PALO-04-07	EUCTR2005-000298-23	prospective	low	-
Park-2013	NCT01476280	retrospective	unclear	-
Rettori-2011	NCT00888303	retrospective	unclear	-
Ryoo-2015	KCT0000581	retrospective	unclear	-
Ryu-2013	KCT0000267	retrospective	unclear	-
Sanchez-Rodriguez-2010	NCT01030614	retrospective	unclear	-
Segelman-2016	EUCTR2009-014717-27	prospective	unclear (either protocol or	-

			study article provides no information about the "time assessment")	
Singla-2010	NCT00108095	retrospective	unclear	-
Sinha-2014	NCT00956215	prospective	high	1
Soga-2015	JPNR-UMIN000007613	prospective	high	2
Song-2017	NCT02480088	retrospective	unclear	-
Tarantino-2015	NCT01189292	prospective	low	-
Tolver-2012	NCT01170780	prospective	low	-
Tsutsumi-2014	JPNR-UMIN000008621	prospective	unclear (either protocol or study article provides no information about the "time assessment")	-
Vallejo-2012	NCT00659945	prospective	high	1
Worni-2008	NCT00619086	retrospective	unclear	-
Yang-2015	NCT02096835	retrospective	unclear	-
Yang-2017	NCT02039427	prospective	high	1
Zhou-2012	ChiCTR-TRC-09000558	prospective	high	2
