# Supplementary material for: Trial registration and selective outcome reporting in 585 clinical trials investigating drugs for prevention of postoperative nausea and vomiting
Source: BMC Anesthesiol. 2021 Oct 19;21:249. doi: 10.1186/s12871-021-01464-w (PMC8524993; doi:10.1186/s12871-021-01464-w)
Supplement: Supplementary file 4 — Additional file 4. List of journals publishing the registered trials (n = 75), with date of adopting the ICMJE policy. [file 12871_2021_1464_MOESM4_ESM.pdf]

Supplementary File 4: List of journals publishing registered trials (n = 75), with date of adopting the ICMJE policy.

Journal	Total (n=75)	list date (M/D/Y) of including ICMJE policy on prospective registration	Publication before or in the year of the list date (n) / *(number of prospective registrations)	Publication after list date (n) / *(number of prospective registrations)
Acta Anaesthesiologica Scandinavica	2/75 (2.6%)	1/1/2010§	1	1
Aesthetic Plastic Surgery	1/75 (1.3%)	7/16/18*	1	0
American Journal of Therapeutics	1/75 (1.3%)	NA	na	na
Anesthesia & Analgesia	4/75 (5.3%)	3/3/16*	4 / *(1)	0
Anesthesiology	2/75 (2.6%)	5/1/2013§	1	1
Anesthesiology and Pain Medicine	1/75 (1.3%)	9/12/11*	0	1
Annals of Surgery	2/75 (2.6%)	7/1/2007§	0	2 / *(1)
Archives of Surgery	1/75 (1.3%)	NA	na	na
BioMed Research International	1/75 (1.3%)	NA	na	na
BMC Anesthesiology	3/75 (4.0%)	pro/retro #	na	na
BMC Cancer	1/75 (1.3%)	pro/retro #	na	na
British Journal of Anaesthesia	8/75 (10.6%)	1/1/2009§	1	7 / *(4)
British Journal of Surgery	1/75 (1.3%)	wo date	na	na
Drug Design, Development and Therapy	1/75 (1.3%)	9/4/14*	0	1 / *(1)
European Journal of Anaesthesiology	2/75 (2.6%)	1/1/2015§	0	2 / *(1)
European Journal of Cancer Care	1/75 (1.3%)	pro/retro #	na	na
European Journal of Cardio-Thoracic Surgery	1/75 (1.3%)	3/24/14*	0	1 / *(1)
European Journal of Clinical Pharmacology	1/75 (1.3%)	12/20/16*	1 / *(1)	0
Frontiers in Medicine	1/75 (1.3%)	5/27/15*	0	1
Head & Neck	1/75 (1.3%)	NA	na	na
Indian Journal of Anaesthesia	1/75 (1.3%)	5/29/18*	1	0
International Journal of Surgery	1/75 (1.3%)	3/10/15*	1	0
Iranian Journal of Medical Sciences	1/75 (1.3%)	1/19/10*	0	1

Journal of Anesthesia	6/75 (8.0%)	4/1/2013§	1	5 / *(3)
Journal of Babol University of Medical Sciences	1/75 (1.3%)	NA	na	na
Journal of Clinical Anesthesia	2/75 (2.6%)	NA	na	na
Journal of International Medical Research	1/75 (1.3%)	pro/retro #	na	na
Journal of Neurosurgical Anesthesiology	1/75 (1.3%)	wo date§	na	na
Journal of the Medical Association of Thailand	2/75 (2.6%)	4/10/19*	2	0
Korean Journal of Anesthesiology	4/75 (5.3%)	7/27/17*	4 / *(2)	0
Minerva Chirurgica	1/75 (1.3%)	NA	na	na
Obesity Surgery	2/75 (2.6%)	7/16/18*	2 / *(1)	0
Pain	1/75 (1.3%)	8/19/15*	1	0
Plastic and Reconstructive Surgery	1/75 (1.3%)	6/2/20*	1 / *(1)	0
PLOS ONE	1/75 (1.3%)	pro/retro #	na	na
Rawal Medical Journal	1/75 (1.3%)	NA	na	na
Surgical Endoscopy	1/75 (1.3%)	7/2/20*	1	0
Surgical Laparoscopy, Endoscopy & Percutaneous Techniques	1/75 (1.3%)	NA	na	na
The Journal of Medical Investigation	1/75 (1.3%)	NA	na	na
The New England Journal of Medicine	1/75 (1.3%)	7/1/2008§	1	0
World Journal of Surgery	2/75 (2.6%)	pro/retro #	na	na
no journal publication (Clinical Study Report)	6/75 (8.0%)	na	na	na

NA (not available), na (not applicable), wo date (without date; the journal endorses trial registration, but does not indicate a date of including ICMJE policy)

*http://www.icmje.org/journals-following-the-icmje-recommendations/ (assessed 8/13/2020)

§information provided on the journal's home page

#pro/retro: The journal requires that clinical trials are prospectively registered in a publicly accessible database. If the trial is not registered, or was registered retrospectively, the reasons for this should be explained (and justifiable).
