# Supplementary material for: Trial registration and selective outcome reporting in 585 clinical trials investigating drugs for prevention of postoperative nausea and vomiting
Source: BMC Anesthesiol. 2021 Oct 19;21:249. doi: 10.1186/s12871-021-01464-w (PMC8524993; doi:10.1186/s12871-021-01464-w)
Supplement: Supplementary file 5 — Additional file 5. Major discrepancies of prospectively registered studies with high risk of selective outcome reporting (SOR). [file 12871_2021_1464_MOESM5_ESM.pdf]

Supplementary File 5: Major discrepancies of high risk SOR studies with prospective study registration

Study-ID	Major discrepancies
DeOliveira-2011	1. Non-significant endpoint defined as non-primary in the published article: **"**The presence of sore throat was less in the dexamethasone 0.1 mg/kg group compared with saline at 24h, but the incidence and severity was not different between dexamethasone groups" [1]<br>2. A new statistically significant efficacy primary outcome was introduced: "The median (IQR) global recovery score (QoR-40) 24h after discharge in the dexamethasone 0.1 mg/kg group was 193 (192–195) which was greater than the score for the dexamethasone 0.05 mg/kg, 179 (175–185) (P = 0.004) or saline, 171 (160–182) groups (P = 0.005).” [3]<br>3. The secondary outcome in the protocol "QoR-40" was the primary outcome in the published study: "The median (IQR) global recovery score (QoR-40) 24h after discharge in the dexamethasone 0.1 mg/kg group was 193 (192–195) which was greater than the score for the dexamethasone 0.05 mg/kg, 179 (175–185) (P = 0.004) or saline, 171 (160–182) groups (P = 0.005)." [4]<br>4. The time assessment in the protocol claims “Sore throat pain at 24 hours [Time Frame: 24 hours]”. The primary outcome in the study time assessment claims “Sore throat assessment 1, 3 and 24h.” [5]
Fahlenkamp-2016	1. The omitted primary outcome of the protocol "the average depth of hypnosis" cannot be evaluated "because the published text contained no results concerning the registered primary outcome" [2]<br>2. The time assessment in the primary outcome in the protocol claims "nausea assessed at 2,6 and 24h after anaesthesia". The primary outcome in the study only claims "early nausea". Non-significant "late nausea" is no longer a primary study outcome ("Late-onset nausea (2-6h and6-24hpost anaesthesia) and vomiting were not affected by anaesthetic technique in a relevant way" [5]
Green-2012	1. The primary outcome in the protocol "incidence of PONV" is a secondary outcome in the article. This is a "non-significant one (which) was omitted or defined as non-primary in the published article", "The incidence of PONV in the post-anaesthesia care unit did not differ nor did the use of rescue medications." [1]<br>2. The new primary outcome in the study is "Complete response from 0-24h". This primary outcome is a non-significant in the article "The aprepitant alone and aprepitant with scopolamine did not differ in complete responses (63% vs 57%, P = 0.57) or net clinical benefit (26% vs 19%, P = 0.38)" [3]
Hu-2017	1. The primary outcome in the protocol "severity of nausea and vomiting" is a secondary outcome in the article. This is a "non-significant one (which) was omitted or defined as non-primary in the published article", "No significant difference was found between the vomiting scores of each group. A significant difference in the nausea score was observed at 0–4 h (P=0.0007) and 24–48 h (P=0.0002) between each group. The nausea severity in Group P7.5 and Group P+D was significantly lower than that in Group P2.5 at 0–4 h (PP2.5–P7.5=0.0159, PP2.5–P+D=0.0003) and 24–48 h (PP2.5–P7.5=0.0032, PP2.5–P+D=0.0032), while the nausea score between Group P7.5 and Group P+D was similar (P=0.3580). No significant difference in the vomiting score was observed at any other intervals, between the three groups (Table 2)" [1]<br>2. The published article provides no information about the protocol primary outcome "time to treatment failure" [2]

Kim-SH-2013	1. The new primary outcome in the study is "need to rescue medication". This primary outcome favoured statistically significant results: "The rescue antiemetic was used less frequently in the palonosetron group than in the other 2 groups etc." [3]<br>2. The published article provides no information about the protocol primary outcome time "73h." [5]
Sinha-2014	1. The primary outcome in the protocol "nausea" is a secondary outcome in the article. This is a "non-significant one (which) was omitted or defined as non-primary in the published article", "table 2 in the article" [1]
Soga-2015	1. The primary outcome in the protocol "PONV" is a secondary outcome in the article. This is a "non-significant one (which) was omitted or defined as non-primary in the published article", "The PONV incidence, complete response rate, nausea score, and VAS score were not significant between the two groups at all time points, i.e., 0–2, 0–24, 0–48, and 0–72 h, during the 72-h period after surgery" [1]<br>2. The new primary outcome in the study is "incidence of vomiting". This primary outcome favoured statistically significant results: "The incidence of vomiting was significantly lower among patients in the NK1 group in comparison to the control group at 0–24 h (0 vs 20 %, respectively; P = 0.023); 0–48 h (0 vs 20 %, respectively; P = 0.023); and 0–72 h (0 vs 30 %, respectively; P = 0.010) (Table 3)." [3]
Vallejo-2012	1. The primary outcome in the protocol "incidence of nausea" is a secondary outcome in the article. This is a "non-significant one (which) was omitted or defined as non-primary in the published article", "The incidence of nausea was not significantly different in the two groups" [1]
Yang-2017	1. The time assessment in the primary outcome in the protocol claims "POST assessed at 1,6 and 24 h after extubation". The primary outcome in the study time assessment only claims "POST assessed at 6 hours after extubation".This time assessment does not favour statistical significant outcomes "Incidences and severities of POST at rest and during swallowing in first 6 hours after extubation were comparable among 4 groups" [5]
Zhou-2012	1. The new primary outcome in the study is "pain". This primary outcome favoured statistically significant results: "After the operation, all patients experienced throat pain. At 2, 4, 8, and 16 h postoperatively, the severity of pain was significantly lower in group D and group DT than in group T (P<0.01 for 2h; P<0.05 for 4, 8, and 16h) (Fig. 6)" [3]<br>2. The published article provides no information about the protocol primary outcome time "73h." [5]

According to studies from Chan et al. [6] and Mathieu et al. [11] major discrepancies between the registered and published outcomes were defined using the following criteria:

1. The registered primary outcome was reported as a secondary outcome in the published article.[1]
2. The registered primary outcome was omitted in the published report. [2]
3. A new primary outcome was introduced in the published article.[3]
4. The published primary outcome was registered as a secondary outcome. [4]
5. The timing of assessment of the registered and published primary outcomes differed.[5]
